# Fatty acid composition analyses of commercially important fish species from the Pearl River Estuary, China

**DOI:** 10.1371/journal.pone.0228276

**Published:** 2020-01-30

**Authors:** Xiyang Zhang, Xi Ning, Xiaoxiao He, Xian Sun, Xinjian Yu, Yuanxiong Cheng, Ri-Qing Yu, Yuping Wu

**Affiliations:** 1 Zhuhai Key Laboratory of Marine Bioresources and Environment, Guangdong Provincial Key Laboratory of Marine Resources and Coastal Engineering, School of Marine Sciences, Sun Yat-Sen University, Zhuhai, China; 2 Southern Marine Science and Engineering Guangdong Laboratory (Zhuhai), Zhuhai, China; 3 The Third Affiliated Hospital of Southern Medical University, Guangzhou, China; 4 Department of Biology, Center for Environment, Biodiversity and Conservation, The University of Texas at Tyler, Tyler, Texas, United States of America; Universiti Sains Malaysia, MALAYSIA

## Abstract

Evaluation of fish nutritional content information could provide essential guidance for seafood consumption and human health protection. This study investigated the lipid contents, fatty acid compositions, and nutritional qualities of 22 commercially important marine fish species from the Pearl River Estuary (PRE), South China Sea. All the analyzed species had a low to moderate lipid content (0.51–7.35% fat), with no significant differences in fatty acid profiles among fishes from different lipid categories (*p* > 0.05). Compared with previous studies from other regions, the examined fish species exhibited higher proportions of saturated fatty acids (SFAs, 39.1 ± 4.00%) and lower contents of polyunsaturated fatty acids (PUFAs, 21.6 ± 5.44%), presumably due to the shifted diet influence from increased diatoms and decreased dinoflagellate over the past decades in the PRE. This study further revealed that there was a significantly negative correlation between the trophic levels and levels of PUFAs in the examined species (Pearson’s *r* = -0.42, *p* = 0.04), likely associated with their differed dietary composition. Considering the health benefit of PUFAs, a few marine fish in PRE with low levels of PUFAs might have no significant contribution to the cardiovascular disease prevention, although fish with different fatty acid profiles most likely contribute differently towards human health. Additional studies are needed in order to comprehensively analyze the nutritional status of fish species in the PRE.

## Introduction

Previous studies revealed an interestingly tight correlation between the low cardiovascular disease occurrence rates in Greenlandic Inuit people and Japanese and their high intake of seafood fat [1–2]. The dynamic coupling and causal relationships have brought increasing attention on the health significance of fatty acids, especially polyunsaturated fatty acids (PUFAs), in human nutrition. Fatty acids are essential for life due to their vital roles as a source of membrane constituents, energy, and metabolic and signaling mediators [3]. PUFAs are fatty acids that contain 18 or more carbon atoms and more than one double bonds. Based on the position of the last double bond relative to the terminal methyl end of the molecule, the PUFAs are further divided into three classes: *n-*3 PUFAs, *n*-6 PUFAs, and *n*-9 PUFAs. Among them, *n*-3 PUFAs and *n*-6 PUFAs are essential fatty acids which can’t be synthesized in mammals. Over the last decades, both epidemiological and experimental studies have provided biomedical evidence to support the protective role of *n*-3 PUFAs. Eicosapentaenoic acid (EPA, C20:5*n*-3) and docosahexaenoic acid (DHA, C22:6*n*-3), especially, play important roles in the prevention of many diseases including cardiovascular diseases, cancers, inflammatory and autoimmune diseases as well as psychiatric and mental illnesses [4–6]. As there was no obvious effect of *n*-6 PUFAs on cardiovascular prevention [7], recommendations for increasing *n*-3 and reducing *n*-6 consumption have been proposed by nutritionists. However, the intake of *n*-3 fatty acids remains to be relatively low in human populations of many countries and the consumption of *n*-6 fatty acids from vegetable oil and animal fats is still increasing [8–9]. The ratios of *n*-6 to *n*-3 fatty acids (*n*-6/*n*-3 ratio) for human diet range from 15:1 to 40:1, which is much higher than the recommended ratio of 4:1 (or lower) in diet for the prevention of cardiovascular diseases by UK Department of Health, and is also higher than the recorded ratios of approximately 1:1 to 2:1 in the hunter-gatherer’s diet of our ancestors [10–11]. This increase in the n-6/n-3 ratio has been presumably associated with the development of numerous diseases [12–14]. Therefore, from a dietary point of view, mitigation of the unfavorable food status by increasing *n*-3 and reducing *n*-6 consumption has been considered as one beneficial nutrition strategy to protect health [15].

Marine fish species are usually characterized by high levels of *n*-3 PUFAs, especially EPA and DHA, which make them a promising source for the supplementation of these nutrients [16]. Generally, the fat nutritional properties of marine fish are largely determined by the advantageous fatty acid profiles that have health-promoting properties. However, the fatty acid compositions of marine fish vary from species to species and can be influenced by many intrinsic and extrinsic factors, such as diet, age, size, and environmental conditions [10]. It is thus crucial to gain information of the nutritional qualities for commercially important fish species in order to evaluate their health benefits on the consumers. Considering that the ratio of *n*-6 to *n*-3 fatty acids (*n*-6/*n*-3 ratio) is the only main parameter for evaluating the human diet, additional indices based on the functional effects of individual fatty acid are warranted to fill the gap on the nutritional property evaluation of marine fish [17].

China has become the largest producer of fish in the world over one decade, although the per capita consumption of fish and fish products (9.66 kg per capita per year) is still relatively low in comparison with many other countries [18–19]. From all Chinese estuarine regions, however, the studies on fatty acid contents and nutritional qualities of various fish species are surprisingly limited, with only one report on the fatty acid composition analyses of twelve marine species in the Zhoushan fishing ground from the East China Sea [18]. The Pearl River Estuary (PRE) is one of the most important fishing grounds in China. It was reported that more than 280 fish species with more than 50 belonging to commercial fishes were found in the PRE fishing ground [20]. Although these commercial species are of growing interest to both aquatic product producers and consumers, there are currently no available fatty acid composition data for the evaluation of their nutritional properties. In view of these facts, the purposes of this study were to analyze the total lipid content, characterize the fatty acid composition, and further evaluate the lipid nutritional quality of the highly consumed marine fish species from the PRE. The selected 22 marine fish species in this study represent roughly half of the commercially important marine fishes, accounting for the majority of the total marine fish production in the PRE. Results of this study would provide beneficial evaluation evidence for both fish production and consumption, and further offer instrumental data for developing public health nutrition programs and strategic policies. Moreover, most of the selected species are also the predominant prey items of the Indo-Pacific humpback dolphins in the PRE, which is the only cetacean species listed in the Grade I National Key Protected Animal in China. Consequently, the information presented here would also contribute to a better understanding of the nutrition status of this protected species in the PRE.

## Materials and methods

### Ethics statement

The research was conducted under the permit of the Department of Agriculture and Rural Affairs of Guangdong Province and the National Natural Science Foundation of China (Permit No. 41576128). The sampling procedure was in compliance with the laws and regulations of this kind of studies in China. All animal procedures were approved by the Animal Ethics Committee of the Sun Yat-Sen University School of Marine Sciences and were carried out in accordance with the Statute on the Administration of Laboratory Animals of China.

### Sample collection and processing

Samples were collected by trawling in the PRE (latitude 21.9215° N ~ 22.5534° N; longitude 113.4590° E ~ 113.8716° E) during the fishing season in 2013. Ten individuals from each fish species were sampled. After collection, samples were immediately placed in a portable cooler and transported directly to the laboratory. Upon arrival, each undamaged individual was selected and the body length and weight of each individual were recorded. All the samples were kept at -80 °C prior to analysis.

### Total lipid extraction

Lipid extraction was performed according to the modified Folch method [21]. Briefly, the whole fish was homogenized with a Waring blender. After that, 1.5 g of homogeneous mixture was homogenized overnight with 30 mL chloroform/methanol mixture (2:1, by vol.), which contained 0.01% butylated hydroxytoluene as the antioxidant. The homogenate was then filtered through a fat-free paper into a 40 mL centrifuge tube. New chloroform/methanol mixture was added to rinse the jar and the filter paper until reaching a final volume of 33 mL. The filtrate is mixed thoroughly with 7 mL of 0.9% sodium chloride solution. After layering, the entire lower phase was collected and filtered through approximately 1 g of anhydrous sodium sulfate. After rinsing three times with approximately 1 mL of chloroform, the filter was then evaporated to a constant weight at ambient temperature under a stream of nitrogen. Methylene chloride was added to make up a final concentration of 100 mg mL^-1^.

### Fatty acids analysis

Fatty acids methyl esters (FAMEs) were obtained from total lipids by acid-catalyzed transesterification [22]. Briefly, an amount of 100 mg of the extracted lipid was dissolved in 1.5 mL dichloromethane and 3 mL methanol-sulfuric solution (200:3 at a volume ratio) and shaken vigorously. The reaction mixture was heated at 100 °C for approximately 1 h and then cooled to room temperature. Subsequently, 3 mL hexyl hydride and 1 mL distilled water were added to the mixture. After vortexing and layering, the entire upper phase was collected. One gram of anhydrous sodium sulfate was added to the collected sample and then stewed for overnight stratification. After filtration, the sample was evaporated until its weigh turned to be constant under a stream of nitrogen. Heptane was added to the sample to make up a final concentration of 50 mg mL^-1^. The qualitative analysis of FAMEs was carried out using a gas chromatograph (Varian CP3800, Varian, Inc., Palo Alto, CA, USA) equipped with a BPX70 fused silica capillary column (length, 50 m; internal diameter, 0.22 mm; film thickness, 0.25 μm) and a flame-ionization detection (FID). The carrier gas was nitrogen and the split ratio was set at 1:100. The temperatures for the injection port and detector were set at 270 °C and 300 °C, respectively. Mass spectra data were processed using STAR-GC3800 software. Fatty acids were identified by comparing their retention time with a mixture of standards containing 40 fatty acids (NU-CHEK PREP, INC.) in this study. Each fatty acid was quantified by calculating its peak area relative to the total peak area. These values are referred to as fatty acid content (%) throughout the paper.

### Nutritional quality indexes of lipids

The nutritional lipid quality of the studied fish was evaluated by five nutritional indicators based on fatty acid compositions. Index of *n*-6/*n*-3 ratio refers to the comparison of the *n*-6 PUFAs over *n*-3 PUFAs. Index of P/S ratio refers to the fraction of the PUFAs over SFAs. Indices of atherogenicity (IA) and thrombogenicity (IT) were estimated according to Ulbricht & Southgate (1991), and the index of hypocholesterolemic/hypercholesterolemic fatty acids (HH) was calculated according to Santos-Silva, Bessa, & Santos-Silva, (2002) with the following formulae:

1. IA = [(C12:0 + (4 × C14:0) + C16:0)]/(MUFAs + *n*-6 PUFAs + *n*-3 PUFAs) [23].
2. IT = (C14:0 + C16:0 +C18:0/[(0.5 × MUFAs) +(0.5 × *n*-6 PUFAs) + (3 × *n*-3 PUFAs) + (*n*-3 PUFAs/ *n*-6 PUFAs)] [23].
3. HH = (C18:1*n*-9 + C18:2*n*-6 + C20:4*n*-6 + C18:3*n*-3 +C20:5*n*-3 + C22:5*n*-3 + C22:6*n*-3)/(C14:0 + C16:0) [24].

### Statistical analysis

Each result shown in Table 1 and 2 is the mean value derived from the analyses of 10 samples from a given species. Fatty acids were grouped as saturated fatty acids (SFAs), monounsaturated fatty acids (MUFAs), and PUFAs for each species to show their composition and overall variations among the fish. Fish were further classified into different categories based on their lipid content [25]. Differences in fatty acid signature among the analyzed fish of different categories were determined by one-way analysis of variance (ANOVA) with Tukey’s test at a 5% significance level (*p* < 0.05) (SPSS Software version 20.0, SPSS Inc., Chicago, IL, USA). Pearson correlation coefficient tests were performed to examine the possible relationships between fatty acid signatures and trophic levels. The trophic level data for the studied fish species were obtained from www.fishbase.org. In order to compare the nutritional quality of the studied marine fishes in the PRE, hierarchical clustering analyses focusing on the five nutritional quality indices (*n*-6/*n*-3, P/S, HH, IA, and IT) in different species were conducted by using Pheatmap package (https://cran.r-project.org/web/packages/pheatmap/index.html, version 1.0.10) in R language. The results were visualized with the presentation of heat maps.

**Table 1:** Fish species (n = 10 for each species) examined in the present study and their lipid contents and trophic levels.

Scientific name	Common name	Trophic level ^a^	Lipid content (g/100 g)	Body length (cm)	Body weight (g)
*Odontamblyopus rubicundus*	Rubicundus eelgoby	3.9 ± 0.5	1.38	18.1 ± 1.9	24.1 ± 6.4
*Sillago sihama*	Silver sillago	3.3 ± 0.1	0.51	18.2 ± 1.3	37.5 ± 5.3
*Selaroidea leptolepis*	Yellow stripe trevally	3.8 ± 0.2	2.10	12.0 ± 2.1	25.4 ± 3.8
*Arius sinensis*	Chinese sea catfish	3.9 ± 0.7	2.70	22.9 ± 2.7	104.0 ± 19.2
*Collichthys lucidus*	Big head croaker	3.6 ± 0.6	5.50	9.7 ± 1.3	22.5 ± 4.2
*Ilisha elongata*	Chinese herring	3.8 ± 0.6	4.84	28.6 ± 4.6	304.5 ± 24.1
*Thryssa kammalensis*	Kammal thryssa	3.4 ± 0.4	7.35	10.4 ± 1.9	19.5 ± 4.3
*Thryssa dussumieri*	Dussumier’s thryssa	2.8 ± 0.2	7.20	8.4 ± 1.4	17.1 ± 3.3
*Larimichthys polyactis*	Yellow croaker	3.7 ± 0.4	3.08	13.5 ± 1.8	70.1 ± 5.9
*Trichiurus lepturus*	Hairtail	4.4 ± 0.4	6.57	51.5 ± 2.2	78.7 ± 8.2
*Pampus argenteus*	Silver pomfret	3.3 ± 0.1	6.60	20.5 ± 2.6	168.8 ± 6.6
*Coilia mystus*	Anchovies	3.2 ± 0.4	6.28	17.1 ± 2.0	16.1 ± 2.2
*Cynoglossus lida*	Roughscale tonguesole	3.2 ± 0.1	2.17	18.7 ± 3.5	93.6 ± 5.3
*Harpadon nehereus*	Bombay duck	4.2 ± 0.7	1.41	21.8 ± 2.9	96.2 ± 4.2
*Johnius belangerii*	Belanger’s croaker	3.3 ± 0.3	6.35	13.9 ± 0.8	48.8 ± 3.8
*Nemipterus virgatus*	Golden threadfin bream	4.0 ± 0.6	2.66	18.8 ± 0.7	76.1 ± 8.7
*Mugil cephalus*	Flathead mullet	2.5 ± 0.2	4.77	17.2 ± 0.4	52.4 ± 3.3
*Siganus fuscessens*	Dusky rabbitfish	2.0 ± 0.1	4.71	12.6 ± 0.5	37.1 ± 5.6
*Branchiostegus albus*	Horsehead tilefish	3.4 ± 0.5	2.31	22.4 ± 3.2	231.5 ± 6.5
*Leiognathus brevirostris*	Shortnose ponyfish	3.0 ± 0.3	6.00	12.5 ± 1.3	27.7 ± 1.7
*Trypauchen vagina*	Burrowing goby	3.5 ± 0.5	3.22	13.8 ± 2.2	12.5 ± 2.4
*Lateolabrax japonicus*	Sea perch	3.1 ± 0.3	1.60	29.5 ± 2.1	228.2 ± 26.4

^*a*^ All data was obtained from the fishbase (www.fishbase.org).

**Table 2:** The fatty acid compositions (% of total fatty acids) of twenty-two marine fish species from the Pearl River Estuary (PRE), China.

**Fatty acids (%)**	***Sillago sihama***	***Odontamblyopus rubicundus***	***Harpadon nehereus***	***Lateolabrax japonicus***	***Selaroidea leptolepis***	***Cynoglossus lida***	***Branchiostegus albus***	***Nemipterus virgatus***	***Arius sinensis***	***Larimichthys polyactis***	***Trypauchen vagina***
C14:0	2.95	2.99	7.47	2.54	5.24	5.15	4.33	3.69	2.1	2.22	3.41
C15:0	0.60	1.50	0.40	0.76	0.97	1.25	0.71	1.29	0.55	0.70	0.87
C16:0	20.9	20.2	26.3	25.2	31.7	20.2	26.1	28.6	33.3	26.1	19.2
C17:0	1.72	2.04	0.78	1.85	1.59	3.07	0.99	2.03	1.26	1.17	2.41
C18:0	8.34	8.24	3.63	9.29	8.24	6.72	7.82	9.96	6.82	8.21	8.62
**SFAs**	**34.5**	**35.0**	**38.6**	**39.7**	**47.7**	**36.4**	**40.0**	**45.6**	**44.0**	**38.4**	**34.5**
C14:1	0.50	0.43	0.44	0.37	0.31	0.40	0.56	0.26	0.20	0.32	0.28
C15:1	0.32	0.36	0.66	0.42	0.13	0.30	0.30	0.19	0.25	0.22	0.51
C16:1*n*-7	14.4	10.8	19.9	8.10	10.7	9.32	8.88	6.06	8.06	9.05	8.93
C17:1	0.94	1.25	0.48	0.98	0.57	1.02	0.91	0.74	0.65	0.63	0.49
C18:1*n*-7	5.90	3.72	2.81	3.93	3.88	4.38	2.70	2.74	2.52	3.12	5.60
C18:1*n*-9	11.2	8.92	13.0	11.7	7.97	8.37	18.8	7.80	22.3	12.7	9.46
C20:1	0.88	1.31	0.97	0.91	0.28	3.96	1.40	0.77	1.02	0.76	0.79
C22:1	0.58	0.42	0.31	0.79	0.38	3.66	0.57	0.41	0.71	0.45	0.61
C24:1	0.11	0.1	0.16	0.15	0.07	0.16	0.28	0.28	0.14	0.4	0.11
**MUFAs**	**34.7**	**27.3**	**38.7**	**27.4**	**24.3**	**31.6**	**34.4**	**19.3**	**35.8**	**27.6**	**26.8**
C18:2*n*-6	1.20	1.16	0.57	1.63	1.05	1.09	0.68	1.2	1.02	0.77	2.81
C18:3*n*-3	0.88	0.55	0.45	0.96	0.62	0.64	0.17	0.38	1.00	0.35	1.02
C18:3*n*-6	0.10	0.12	0.08	0.11	0.18	0.18	0.06	0.1	0.07	0.09	0.32
C18:4*n*-3	1.84	1.70	0.09	0.84	0.53	ND	1.16	ND	0.78	0.12	1.96
C20:2*n*-6	0.56	0.56	0.11	0.41	0.12	0.04	0.30	0.27	0.22	0.16	0.28
C20:3*n*-3	2.07	5.38	1.43	2.72	1.82	1.67	1.95	2.57	1.67	2.39	2.25
C20:4*n*-6	0.12	0.06	0.03	0.06	ND	0.12	ND	ND	0.08	ND	0.05
C20:5*n*-	8.94	4.32	5.73	5.10	5.74	8.33	2.04	4.73	4.76	4.40	8.09
C22:4	0.25	0.26	0.20	0.15	0.21	0.44	0.07	0.16	0.23	0.13	0.53
C22:5*n*-3	3.31	3.45	0.94	2.13	1.34	2.61	1.84	1.66	1.14	1.17	3.92
C22:5*n*-6	0.52	1.83	0.33	0.05	0.56	0.61	0.63	1.11	0.40	0.7	0.54
C22:6*n*-3	4.15	8.2	5.25	9.16	7.12	7.68	6.44	14.21	3.31	9.6	6.61
**PUFAs**	**23.9**	**27.6**	**15.2**	**23.3**	**19.3**	**23.4**	**15.3**	**26.4**	**14.7**	**19.9**	**28.4**
**Total**	93.2	89.8	92.5	90.4	91.3	91.4	89.7	91.2	94.5	85.9	89.7
**Fatty acids (%)**	***Siganus fuscessens***	***Mugil cephalus***	***Ilisha elongata***	***Collichthys lucidus***	***Leiognathus brevirostris***	***Coilia mystus***	***Johnius belangerii***	***Trichiurus lepturus***	***Pampus argenteus***	***Thryssa dussumieri***	***Thryssa kammalensis***
C14:0	9.27	6.12	3.05	2.09	4.39	3.91	2.34	5.28	5.43	7.86	2.61
C15:0	1.61	0.62	0.5	0.41	0.81	0.44	0.78	0.49	1.16	0.7	0.62
C16:0	24.9	26.0	23.4	24.5	25.0	26.0	24.7	27.3	26.6	28.5	27.3
C17:0	1.26	0.63	0.82	0.77	1.20	0.94	1.38	0.78	1.88	0.62	1.06
C18:0	5.56	5.59	5.76	5.55	5.96	3.87	5.98	5.92	8.90	6.03	6.34
**SFAs**	**42.6**	**39.0**	**33.5**	**33.4**	**37.4**	**35.1**	**35.2**	**39.8**	**44.0**	**43.7**	**37.9**
C14:1	0.59	0.08	0.27	0.20	0.49	0.25	0.28	0.47	0.32	0.29	0.25
C15:1	0.23	0.10	0.11	0.09	0.24	0.33	0.21	0.12	0.91	ND	0.09
C16:1*n*-7	11.7	11.8	7.06	11.1	11.2	10.3	15.5	8.68	6.91	10.2	6.79
C17:1	0.57	0.21	0.55	0.67	0.68	4.46	0.85	0.51	0.62	0.10	0.69
C18:1*n*-7	2.77	5.86	3.38	3.26	3.82	14.91	4.22	2.51	2.00	6.83	2.76
C18:1*n*-9	5.96	11.2	17.3	20.2	13.1	14.1	11.6	18.5	13.7	13.0	16.7
C20:1	0.73	0.29	0.82	0.97	1.49	1.06	1.88	0.53	1.93	0.46	0.52
C22:1	1.31	0.21	0.86	0.68	0.76	0.38	0.58	0.50	1.83	ND	0.54
C24:1	0.14	0.06	0.22	0.11	0.10	0.23	0.27	ND	0.83	0.12	0.45
**MUFAs**	**24.0**	**29.8**	**30.6**	**37.3**	**31.9**	**46.0**	**35.4**	**31.8**	**29.0**	**31.0**	**28.7**
C18:2*n*-6	1.89	6.21	1.04	0.84	1.19	0.93	1.06	0.6	0.84	1.01	0.83
C18:3*n*-3	1.18	1.81	0.95	0.8	1.31	1.03	0.85	0.74	0.3	0.46	0.49
C18:3*n*-6	0.23	0.53	0.11	0.11	0.17	0.09	0.11	0.13	0.04	0.13	0.09
C18:4*n*-3	1.06	ND	0.9	0.68	1.72	ND	0.27	ND	ND	ND	0.48
C20:2*n*-6	0.23	0.17	0.25	0.18	0.43	0.19	0.27	ND	0.21	0.2	0.17
C20:3*n*-3	1.57	0.15	1.24	1.02	1.28	1.03	2.65	0.76	1.98	0.13	2.33
C20:4*n*-6	ND	1.4	ND	0.12	0.11	0.03	0.05	ND	ND	1.33	ND
C20:5*n*-3	2.97	8.23	8.11	7.17	6.76	3.73	6.55	2.44	3.09	9.1	5.58
C22:4	0.24	0.32	0.3	0.38	0.42	0.17	0.25	0.13	0.13	0.29	0.15
C22:5*n*-3	3.95	2	0.96	0.89	1.33	0.91	1.08	0.75	1.2	1.58	0.74
C22:5*n*-6	1.1	ND	0.31	0.25	0.62	0.42	0.69	0.2	1.01	ND	0.47
C22:6*n*-3	11.15	5.35	7.83	5.11	5.99	6.52	7.17	4.54	10.95	7.34	10.1
**PUFAs**	**25.6**	**26.1**	**22.0**	**17.6**	**21.3**	**15.0**	**21.0**	**10.3**	**19.7**	**21.6**	**21.4**
**Total**	92.2	94.9	86.1	88.2	90.6	96.1	91.6	81.9	92.8	96.3	88.1

SFAs: saturated fatty acids; MUFAs: monounsaturated fatty acids; PUFAs: polyunsaturated fatty acids; EPA: eicosapentaenoic acid; DPA: docosapentaenoic acid; DHA: docosahexaenoic acid; IA: index of atherogenicity; IT: index of thrombogenicity, HH: hypocholesterolemic/hypercholesterolemic ratio; n-6/n-3: *n*-6 PUFAs/*n*-3 PUFAs ratio; P/S: PUFAs/SFAs ratio. ND: not detected. Results represent mean values (n = 10).

## Results and discussion

### Lipid content

The crude lipid content of the 22 marine fish species is listed in Table 1. The lipid content, expressed on a wet weight basis, varied from 0.51 g/100 g for *Sillago sihama* to 7.35 g/100 g for *Thryssa kammalensis*. According to the Ackman Standard, fish can be classified into four categories based on their fat content: lean fish (< 2% fat), low fat (2–4% fat), medium fat fish (4–8% fat), and high fat fish (> 8% fat) [25]. Based on this classification system, eleven were considered to be medium fat fish (11 in 22), seven were found to be low-fat fish, and four could be classified as lean fish (Table 1). No high-fat fish were found in our study. Thus, most of the fish species investigated in this study contained a low to moderate lipid content. Such lipid contents were comparable to those of marine fish (0.58–7.83 g/100 g) collected from the East China Sea [18], but lower than those of marine fish (1.19–11.9 g/100 g) in the Southern Yellow Sea [26]. It should be noted that although lipid content reported herein could be mainly attributed to differences among the species, the fat content might be also influenced by multiple factors, including geographical origin, catch season, diet, reproductive stage, and age variation [18, 27].

### Fatty acid profiles

Fatty acid compositions of sampled fish species are summarized in Table 2. Overall, this study identified twenty-six fatty acids with the minimum proportion which exceeded 0.1% of the total fatty acids, composed of C14:0 to C22:6*n*-3. The 26 fatty acids reported here constituted about 81.9%–96.3% of the total fatty acids. Wide variations in the fatty acid composition were shown among all fish species (Table 2). Fatty acids were categorized into SFAs, which accounted for 33.4% to 47.7% of total fatty acids in all studied species; MUFAs, which constituted 19.3% to 46.0%; and PUFAs, which comprised 10.3% to 28.4%. In general, most of the studied species (17 in 22) contained a higher proportion of SFAs than that of MUFAs and PUFAs. The exception was the proportion with *Sillago sihama*, *Collichthys lucidus*, *Coilia mystus*, *Harpadon nehereus* and *Johnius belangerii*, which followed a variant pattern with MUFAs > SFAs > PUFAs.

**Fig 1:**
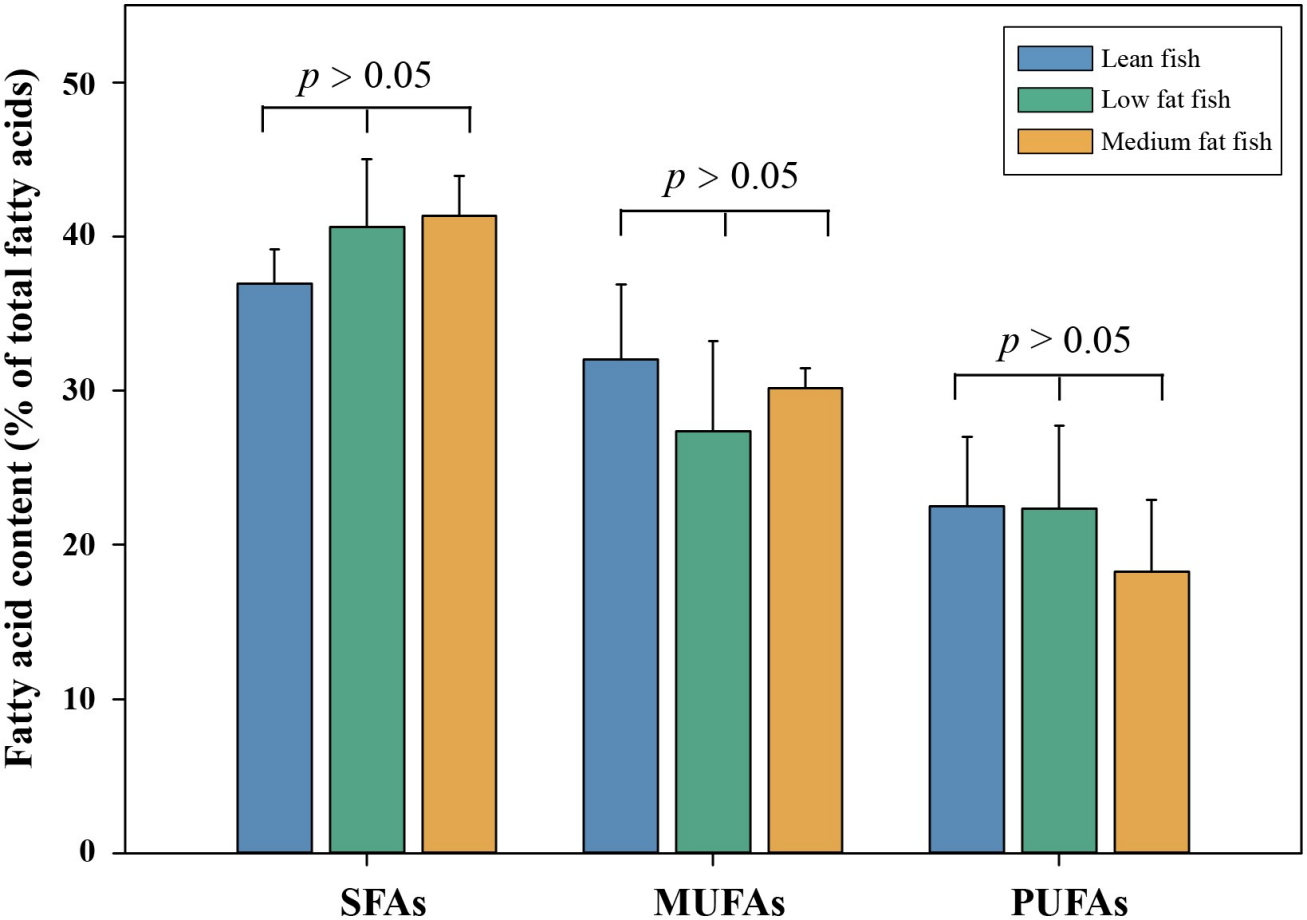
Differences in fatty acid signatures among the analyzed fish from different lipid classes.

There were no significant differences of fatty acid profiles among fishes in different lipid classes (One-way ANOVA, *p* > 0.05, Fig 1). These outcomes were inconsistent with the fatty acid signatures obtained from the previous studies showing that, PUFAs changed in a negative association with the lipid content and MUFAs tended to increase when the lipid content increased [8, 28]. Previous studies revealed that marine fish species were typically characterized by high percentage share of PUFAs [8, 9]. However, the levels of PUFAs in our investigated species were found to be lower than those values reported previously [8, 18, 29]. It is well known that dietary composition, especially the diet’s fatty acid profile, exerts a qualitative and quantitative impact on the fatty acid composition of fish lipids [30]. Thus, the different pattern of fatty acid signatures indicated in this study might largely be influenced by the diet variations. Further investigations should be conducted to obtain additional data regarding the correlation between fatty acid profiles and total lipid content.

#### Saturated fatty acids (SFAs)

Palmitic acid (C16:0), contributing approximately to 65% of the total SFAs, was the predominant SFA in all the examined fish. This proportion is in accord with that found in the previous studies on marine fish species from other regions [8, 31]. Of the overall detected results, *Arius sinensis* contained the greatest, while *Trypauchen vagina* had the smallest proportions of palmitic acid. Stearic acid (C18:0), ranging from 3.63% to 9.96%, was the second most abundant fatty acid identified in all the fishes. Especially noted, the myristic acid (C14:0), which promote hypercholesterolemia, was detected at a relatively high concentration in the examined species compared with previous studies demonstrating a negative factor in their consumption [10, 32]. As SFAs with odd carbon numbers, pentadecanoic acid (C15:0) and heptadecanoic acid (C17:0) were present in small levels. It is generally believed that the consumption of SFAs is associated with a high risk of coronary heart disease and increasing LDL-cholesterol [33]. Thus, the lipid composition of *Collichthys lucidus* with lowest SFAs in our study seems to be healthier than the others, whereas the *Selaroidea leptolepis* with the highest amount of SFAs might be not suitable for the prevention of cardiovascular disease.

#### Monounsaturated fatty acids (MUFAs)

The percentages of MUFAs present in the total lipids of the fish species varied over a wide range (Table 2). The MUFAs in *Coilia mystus* constituted nearly half of the total fatty acids (46.0%), whereas *Nemipterus virgatus* shared relatively low proportions of MUFAs (19.3%). Among all the species tested, the major MUFAs were palmitoleic acid (C16:1*n*-7, 6.06% in *Nemipterus virgatus* to19.9% in *Harpadon nehereus*), vaccenic acid (C18:1*n*-7, 2.00% in *Pampus argenteus* to 14.9% in *Coilia mystus*) and oleic acid (C18:1*n*-9, 5.96% in *Siganus fuscessens* to 22.3% in *Arius sinensis*). In comparison with this study, Li et al. (2011) reported a higher content of oleic acid but a lower content of palmitoleic acid in marine fish species from the East China Sea. The quantity of other fatty acids in this group identified was generally below 1%.

#### Polyunsaturated fatty acids (PUFAs)

The proportion of PUFAs found in the fish species ranged from 10.3% in *Trichiurus lepturus* to 28.4% in *Trypauchen vagina* (Table 2). Among the detected 12 PUFAs, the predominant fatty acids were eicosatrienoic acid (C20:3*n*-3, 0.13% in *Thryssa dussumieri* to 5.38% in *Odontamblyopus rubicundus*), eicosapentaenoic acid (EPA, 2.04% in *Branchiostegus albus* to 9.10% in *Thryssa dussumieri*), docosapentaenoic acid (DPA, C22:5*n*-3, 0.74% in *Thryssa kammalensis* to 3.95% in *Siganus fuscessens*), and docosahexaenoic acid (DHA, 3.31% in *Arius sinensis* to 14.2% in *Nemipterus virgatus*). All species analyzed in this study contained high levels of *n*-3 PUFAs (9.23%-23.9%), but had low levels of total *n*-6 PUFAs (0.93%-8.31%), which were in accordance with previous studies on the fatty acid composition of other marine fishes. The EPA and DHA represented the most abundant *n*-3 PUFA, together accounting for 53.1% of total PUFAs in *Odontamblyopus rubicundus* to 88.3% in *Thryssa dussumieri*. This could be explained by the fact that marine fishes presumably feed predominantly on phytoplankton. Lipids of phytoplankton are generally rich in *n*-3 PUFAs (especially EPA and DHA) and poor in *n*-6 PUFAs such as linoleic and arachidonic acid [34].

Compared with the marine fish species in other regions of the world, which generally contained high PUFAs (e.g., 30–40%), the average proportions of PUFAs for the studied species (20.9 ± 4.61%) in the PRE were much lower (Table 3). Especially the ratios were less than half of the proportions in the European marine fish species from the Adriatic Sea and Polish waters [31, 35]. Moreover, PUFAs of the examined species were among the lowest when compared with those from other fishing grounds in China. PUFAs in marine fishes from Zhoushan fishing ground, the biggest fishing ground in China, accounted for about 28.4% of the total fatty acids [18]. In the Minnan-Taiwan fishing ground, 36.7% of the total fat was PUFAs [36]. Further analyses revealed that EPA and DPA contents in the analyzed species were similar to those observed in previous studies, whereas the DHA contents were substantially low when compared with those from other regions (Table 3).

**Table 3:** Comparison of fatty acid profiles in marine fish species from different regions around the world.

Number of Species	Locations	SFAs	MUFAs	PUFAs	EPA	DPA	DHA	Reference
**23**	**Pearl River Estuary**	**38.9 ± 4.07**	**31.1 ± 5.61**	**20.9 ± 4.61**	**5.72 ± 2.12**	**1.77 ± 1.01**	**7.44 ± 2.55**	**This study**
12	East China Sea	34.5 ± 2.76	31.0 ± 4.10	28.4 ± 5.97	6.81 ± 1.46	2.48 ± 1.21	14.8 ± 3.98	[18]
6	Minnan-Taiwan fishing ground	36.8 ± 1.05	26.6 ± 2.74	36.7 ± 2.41	2.87 ± 0.48	2.54 ± 0.32	20.1 ± 2.10	[36]
8	The northwest Pacific Ocean	25.2 ± 3.73	31.9 ± 12.2	39.6 ± 10.4	10.8 ± 2.01	1.57 ± 0.71	20.1 ± 7.75	[8]
4	Brazil	25.4 ± 6.52	31.1 ± 17.1	31.5 ± 11.7	7.85 ± 1.67	1.60 ± 0.92	19.2 ± 9.73	[10]
4	Panama	39.8 ± 1.72	31.8 ± 1.86	28.3 ± 3.57	6.24 ± 0.87	1.46 ± 0.23	15.3 ± 3.17	[29]
6	Adriatic Sea	31.7 ± 3.01	16.7 ± 4.26	49.9 ± 4.69	8.53 ± 1.64	2.68 ± 2.39	31.3 ± 8.24	[35]
8	Egypt	25.6 ± 2.06	48.3 ± 6.97	26.1 ± 6.61	1.15 ± 0.90	0.92 ± 0.55	10.3 ± 5.69	[42]
11	Queensland	31.9 ± 2.15	17.6 ± 2.51	42.5 ± 2.92	4.09 ± 0.55	3.36 ± 0.58	17.7 ± 3.40	[43]
9	Polish	27.7 ± 6.54	27.4 ± 13.4	45.0 ± 17.9	8.74 ± 9.08	2.46 ± 1.88	20.1 ± 16.2	[31]
8	Turkey	30.5 ± 5.05	21.1 ± 5.80	36.1 ± 8.14	5.40 ± 1.09	NA	21.8 ± 9.18	[16]
8	Mediterranean and Black Seas	31.3 ± 3.50	20.7 ± 4.66	34.8 ± 7.05	6.95 ± 2.55	NA	21.1 ± 8.41	[44]
11	Southern Italy	43.6 ± 3.47	24.9 ± 4.74	31.5 ± 2.28	6.82 ± 0.87	NA	13.8 ± 2.42	[9]
10	India	37.1 ± 2.07	23.7 ± 1.18	32.3 ± 1.48	4.28 ± 0.56	2.74 ± 0.83	3.08 ± 0.86	[45]
6	Black Sea	31.3 ± 2.49	28.4 ± 4.60	27.0 ± 8.01	6.30 ± 2.19	NA	14.5 ± 7.47	[46]

SFAs: saturated fatty acids; MUFAs: monounsaturated fatty acids; PUFAs: polyunsaturated fatty acids; EPA: eicosapentaenoic acid; DPA: docosapentaenoic acid; DHA: docosahexaenoic acid; NA: not analysed. Results represent mean ± SD.

Although both freshwater and marine fish could synthesize PUFAs, such as EPA and DHA, through enzymatic pathways characterized by sequential desaturation and elongation actions, marine species typically present low enzymatic activity and depend almost completely on their diet to obtain the main long-chain *n*-3 fatty acids (e.g. EPA and DHA) [32, 37]. Thus, low fish PUFAs contents in the present study, especially DHA, were likely associated with their dietary composition. As the primary producer in the marine ecosystem, marine microalgae provide the food base which supports the entire marine animal populations. They are also the most important sources of marine bioactive materials, such as EPA and DHA [38]. During the past three decades, the diversity of marine microalgae in the PRE has declined by 37.3% due to environmental degradation [39]. The species composition has also undergone a dramatic change, with the proportion of diatoms increased from 70.1% in the 1980s to 81.0% in the 2010s, whereas the proportion of dinoflagellate reduced from 21.4% to 8.5% during this period [39]. Diatoms are characterized by relatively high values of C14:0, C16:0, C16:1*n*-7, C16:3*n*-4, and C20:5*n*-3 (EPA), while dinoflagellates are rich in PUFAs, especially DHA [40]. Thus, presumably the low PUFAs of fishes investigated in the PRE were associated with the diversity change of marine microalgae, especially the decline of dinoflagellates during the past several decades.

### Relationships between trophic levels and fatty acid profiles

**Fig 2:**
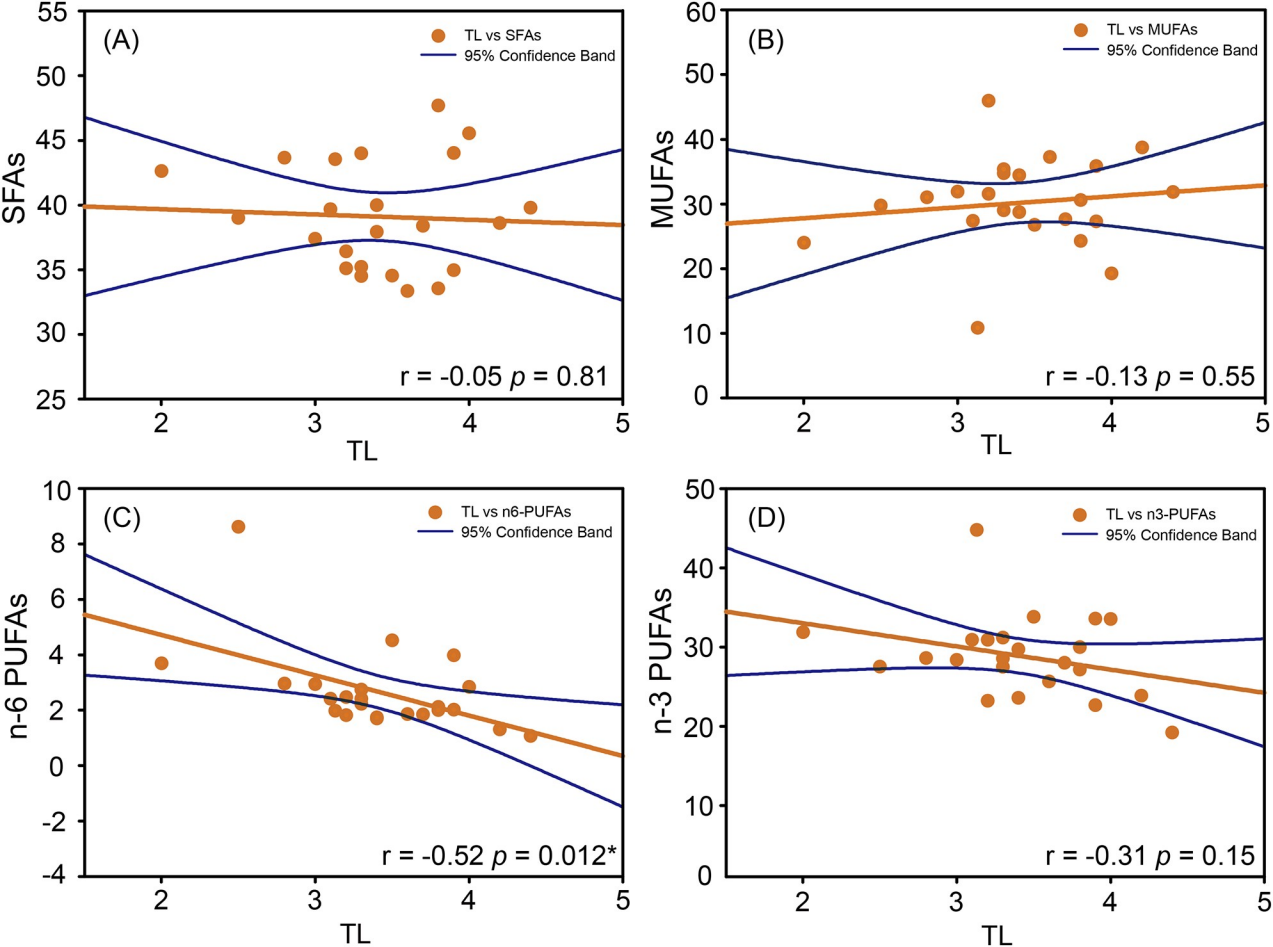
Relationships between trophic levels (TL) and saturated fatty acids (SFAs), monounsaturated fatty acids (MUFAs), *n-*6 PUFAs and *n-*3 PUFAs of the marine fish species from the PRE, China.

The Pearson correlation analysis was used to explore the potential effects of trophic levels on the fatty acid composition of fish species in the PRE. Our results showed no significant relationships between the trophic levels and SFAs and MUFAs (Fig 2A and 2B). However, *n*-6 PUFAs significantly decreased with the increase of trophic levels in the studied species (Fig 2C). A weakly negative correlation pattern was also observed between *n*-3 PUFAs and trophic levels (Fig 2D, *p* = 0.15). It assumes that the fish at low trophic level primarily fed on phytoplankton while the diets of these high trophic level of fish were dominated by small fish and crustaceans. The phytoplankton typically contained higher levels of PUFAs, especially *n*-6 PUFAs, than small fish and crustaceans (the main food sources of those higher trophic level fish species) [34]. Similar results were also observed by Li et al. (2011), showing that the omnivorous fishes with trophic levels below 3.30 had higher total PUFA, *n*-3 PUFA, and EPA + DHA than the carnivorous fish (trophic level > 3.47).

### Nutritional quality indices

**Fig 3:**
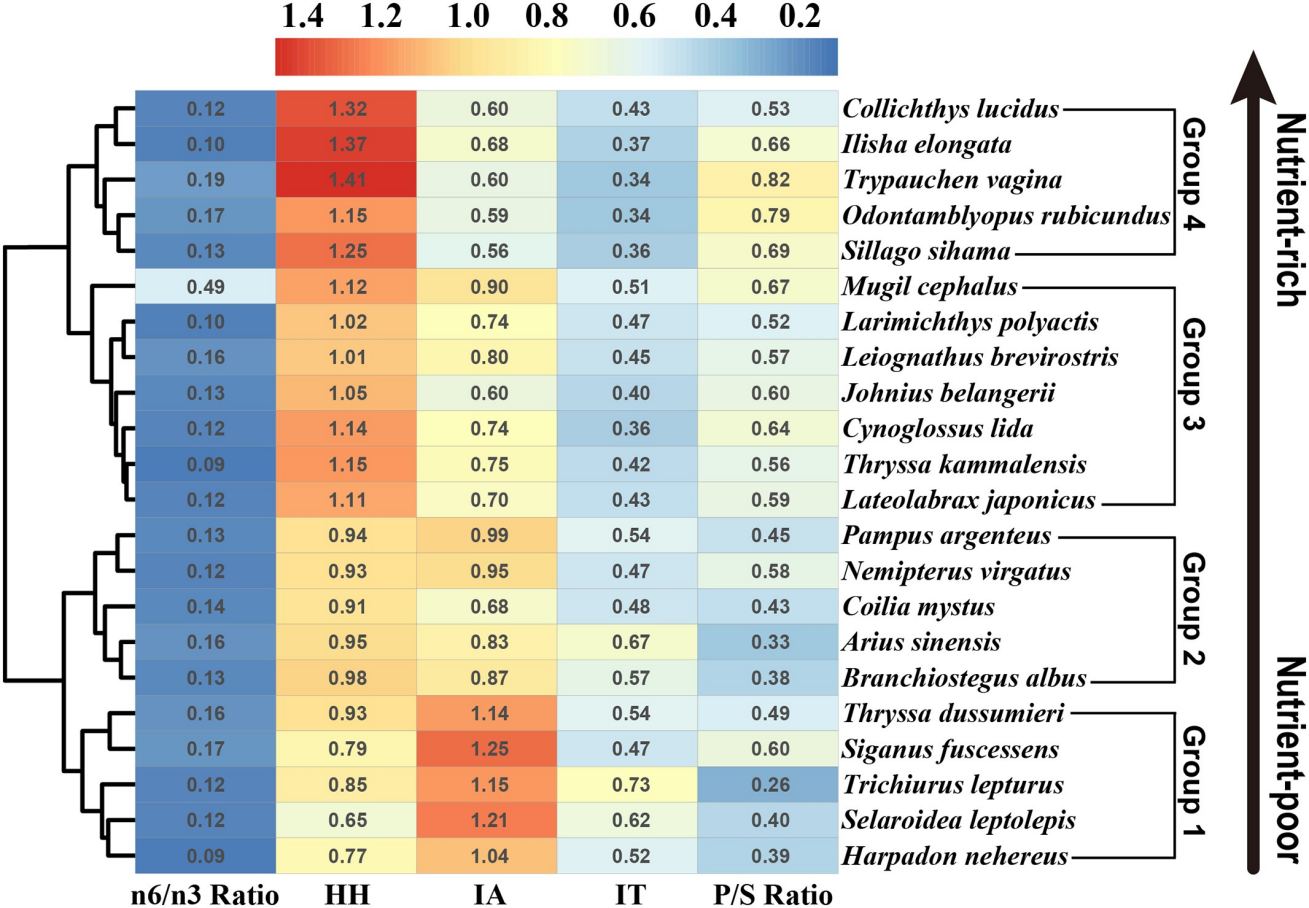
Hierarchical clustering analyses of the selected marine fish species based on the five nutritional quality indices. n-6/n-3 Ratio: *n*-6 PUFAs/*n*-3 PUFAs ratio; HH: hypocholesterolemic/hypercholesterolemic ratio; IA: index of atherogenicity; IT: index of thrombogenicity, P/S Ratio: PUFAs/SFAs ratio.

Fish with different fatty acid profiles most likely contribute differently towards human health. These benefits can be evaluated using the fat quality indices, such as *n*-6/*n*-3 ratio, P/S ratio, IA, IT and HH index [11, 23, 24]. The nutritional qualities of lipid profiles from this study and related indices are showed in Fig 3.

The *n*-6/*n*-3 ratio is considered to be a useful indicator for determining the quality of fat, considering that a lower human dietary *n*-6/*n*-3 fatty acid ratio helps to prevent coronary heart disease by reducing cholesterol in the blood, whereas increased levels of this ratio may promote cardiovascular disease [6]. A ratio value below 4.0 in a diet was recommended for cardiovascular disease prevention by the UK Department of Health [11]. In this study, ratios of *n*-6/*n*-3 PUFAs in the fish species ranged from 0.09 in *Thryssa kammalensis* to 0.49 in *Mugil cephalus*, generally lower than the recommended value from the UK Department of Health, suggesting that these species may be beneficial for human health. The ratio of PUFAs to SFAs is another index commonly used to assess the nutritional value of fats. Compared with the *n*-6 and *n*-3 series fatty acids, the P/S ratio reflects both the effects of PUFAs and SFAs. Foods that have a P/S ratio below 0.45 have been considered undesirable for the human diet because of their potential to increase cholesterol in the blood [11]. In this study, P/S ratios ranged from 0.26 in *Trichiurus lepturus* to 0.79 in *Odontamblyopus rubicundus*, which were lower than those in marine fish species from other studies [10, 16]. The lower P/S ratios in *Selaroidea leptolepis*, *Arius sinensis*, *Trichiurus lepturus*, *Coilia mystus*, *Harpadon nehereus* and *Branchiostegus albus* in this study could be related to the lower PUFAs amount (10.3%-19.3%) present in these species.

Generally, the ratio of P/S or *n*-6/*n*-3 fails to reflect the effects of MUFAs. Without considering the multiple and different functional effects of SFAs and PUFAs, evaluating the nutritional value of fat by only comparing the P/S ratio or *n*-6/*n*-3 ratio might lead to simplistic dietary advice [23–24]. Thus, the IA, IT and HH index based on functional effects of fatty acids were also employed in this study to conduct a comprehensive evaluation of the nutritional quality of the marine fish species from the PRE. The indices of IA and IT are related to pro- and anti-atherogenic and pro- and antithrombogenic fatty acids [23]. High IA and IT values might stimulate platelet aggregation and subsequent thrombus and atheroma formation in the cardiovascular system [32]. Thus, lower IA and IT values are desirable to prevent cardiovascular disorders. The HH index refers to the ratio of hypocholesterolemic over hypercholesterolemic fatty acids and is related with the specific effects of fatty acids on cholesterol metabolism [41]. In contrast to IA and IT, higher HH values are considered more beneficial to human health. Among fish species analyzed, the IA values ranged from the lowest 0.56 in *Sillago sihama* to the highest 1.25 in *Siganus fuscessens*. The IT values ranged from 0.38 in *Trypauchen vagina* to 0.73 in *Trichiurus lepturus*. The average IA and IT values (0.83 and 0.48, respectively) in this study were higher than those reported in other marine species [10, 32, 41]. The HH ratios ranged between 0.65 in *Selaroidea leptolepis* and 1.41 in *Trypauchen vagina*. These ratio results were lower than those of marine fish species from other studying locations [10, 32,41].

Hierarchical clustering analyses based on the five nutritional quality indices (*n*-6/*n*-3, P/S, HH, IA, and IT) were further used to compare the nutritional quality of the studied marine fishes in the PRE. Fish species were clustered into four groups. There was an increase in the nutrient quality of fishes from group 1 to group 4 (Fig 3). Thus, compared with the fish species in group 1 to group 3, *Collichthys lucidus*, *Ilisha elongata*, *Trypauchen vagina*, *Odontamblyopus rubicundus* and *Sillago sihama* from group 4 are more desirable for human consumption in terms of beneficial fats. Compared with the marine fishes from other regions, lower indices of HH and P/S ratios and higher IA and IT indices of fish in this study suggested that marine fish species from the PRE might contain a poor lipid composition with regard to the nutritional purposes. This was in accordance with the conclusion from Usydus et al. (2011), showing that the marine fish species imported from China and Vietnam were characterized by low contents of EPA and DHA, and the consumption of these fish species might have no significant meaning for coronary heart disease prevention.

## Conclusion

This study provided us the first description of the fatty acid profiles and lipid nutritional qualities of the economically important fish species in the PRE, China. Our results showed that fish species from the PRE were generally characterized by high contents of SFAs and low contents of PUFAs, which might be related to the dietary increase of diatoms and the decline of dinoflagellate during the past several decades. PUFAs, especially *n*-6 PUFAs, in the studied species showed a significantly negative relationship with their trophic levels, presumably because the low trophic level of fish primarily fed on phytoplankton which were rich in PUFAs. The study indicated that there were considerable variations in the lipid nutrition in examined fish species from the PRE. Among them, *Collichthys lucidus*, *Ilisha elongata*, *Trypauchen vagina*, *Odontamblyopus rubicundus* and *Sillago sihama*, which contain better nutritional values in terms of P/S ratio, IA, IT, and HH than others, may be more suitable for people who prefer to consume PUFAs-rich fish from this region. In perspective of cardiovascular disease prevention, some fish species, especially those categorized in group 1, from the PRE might have no significant contribution for this purposes due to their poor lipid nutrition quality indices. However, it should be noted that the nutritional benefits of fish stem not only from their fatty acid profiles, but also from the richness in essential and nutritious compounds (e.g., easily digestible proteins, vitamins and polysaccharides). Furthermore, other than cardiovascular disease prevention, variety of additional health-promoting properties might also be highly related with fish consumption. Therefore, further studies are needed in order to make a comprehensive evaluation of the nutritional qualities of the fish species from the PRE.

## References

[1] Dyerberg J, Bang H, Hjørne N. Fatty acid composition of the plasma lipids in Greenland Eskimos. Am. J. Clin. Nutr. 1975; 28(9):958–966. 10.1093/ajcn/28.9.958 1163480

[2] Kagawa Y, Nishazawa M, Suzuki M, Miyatake T, Hamaoto T, Goto K, et al Eicosapolyenoic acids of serum lipids of Japanese islanders with low incidence of cardiovascular diseases. J. Nutr. Sci. Vitaminol. 1982; 28(4):441–453. 10.3177/jnsv.28.441 7175583

[3] Pereira DM, Valentão P, Teixeira N, Andrade PB. Amino acids, fatty acids and sterols profile of some marine organisms from Portuguese waters. Food Chem. 2013;141(3):2412–2417. 10.1016/j.foodchem.2013.04.120 23870975

[4] Berquin IM, Edwards IJ, Chen YQ. Multi-targeted therapy of cancer by omega-3 fatty acids. Cancer Lett. 2008; 269(2):363–377. 10.1016/j.canlet.2008.03.044 18479809 PMC2572135

[5] Peet M, Stokes C. Omega-3 fatty acids in the treatment of psychiatric disorders. Drugs. 2005; 65(8):1051–1059. 10.2165/00003495-200565080-00002 15907142

[6] Simopoulos AP. Omega-3 fatty acids in inflammation and autoimmune diseases. J. Am. Coll. Nutr. 2002; 21(6):495–505. 10.1080/07315724.2002.10719248 12480795

[7] Russo GL. Dietary n-6 and n-3 polyunsaturated fatty acids: from biochemistry to clinical implications in cardiovascular prevention. Biochem. Pharmacol. 2009;77(6):937–946. 10.1016/j.bcp.2008.10.020 19022225

[8] Huynh MD, Kitts DD. Evaluating nutritional quality of pacific fish species from fatty acid signatures. Food Chem. 2009; 114(3):912–918.

[9] Prato E, Biandolino F. Total lipid content and fatty acid composition of commercially important fish species from the Mediterranean, Mar Grande Sea. Food Chem. 2012; 131(4):1233–1239.

[10] Fernandes CE, da Silva Vasconcelos MA, de Almeida Ribeiro M, Sarubbo LA, Andrade SAC, de Melo Filho AB. Nutritional and lipid profiles in marine fish species from Brazil. Food Chem. 2014; 160:67–71. 10.1016/j.foodchem.2014.03.055 24799210

[11] Hmso U. Nutritional aspects of cardiovascular disease (report on health and social subjects No. 46). London: HMSO; 1994. 7863112

[12] Simopoulos AP. The importance of the ratio of omega-6/omega-3 essential fatty acids. Biomed Pharmacother. 2002; 56(8):365–379. 10.1016/s0753-3322(02)00253-6 12442909

[13] El-Badry AM, Graf R, Clavien PA. Omega 3-omega 6: what is right for the liver? J Hepatol. 2007; 47(5):718–725. 10.1016/j.jhep.2007.08.005 17869370

[14] Zhu H, Fan C, Xu F, Tian C, Zhang F, Qi K. Dietary fish oil n-3 polyunsaturated fatty acids and alpha-linolenic acid differently affect brain accretion of docosahexaenoic acid and expression of desaturases and sterol regulatory element-binding protein 1 in mice. J Nutr Biochem. 2010; 21(10):954–960. 10.1016/j.jnutbio.2009.07.011 19954955

[15] Kris-Etherton P, Fleming J, Harris WS. The debate about n-6 polyunsaturated fatty acid recommendations for cardiovascular health. J Acad Nutr Diet. 2010; 110(2):201–204. 10.1016/j.jada.2009.12.006 20102846

[16] Özogul Y, Özogul F, Alagoz S. Fatty acid profiles and fat contents of commercially important seawater and freshwater fish species of Turkey: A comparative study. Food Chem. 2007; 103(1):217–23.

[17] Griffin BA. How relevant is the ratio of dietary n-6 to n-3 polyunsaturated fatty acids to cardiovascular disease risk? Evidence from the OPTILIP study. Curr. Opin. Lipidol. 2008;19(1):57–62. 10.1097/MOL.0b013e3282f2e2a8 18196988

[18] Li G, Sinclair AJ, Li D. Comparison of lipid content and fatty acid composition in the edible meat of wild and cultured freshwater and marine fish and shrimps from China. J. Agric. Food Chem. 2011; 59(5):1871–1881. 10.1021/jf104154q 21291233

[19] Needham S, Funge-Smith S. The consumption of fish and fish products in the Asia-Pacific region based on household surveys. FAO Regional Office for Asia and the Pacific, Bangkok, Thailand RAP Publication 2015;12.

[20] Li Y. Study on fish community structure in the Pearl River Estuary waters. 2008. Master’s thesis. Guangdong Ocean University. China.

[21] Folch J, Lees M, Sloane Stanley G. A simple method for the isolation and purification of total lipids from animal tissues. J. Biol. Chem. 1957; 226(1):497–509. 13428781

[22] Christie WW, Han X. Lipid Analysis: Isolation, Separation, Identification, and Structural Analysis of Lipids. Fourth edition: Oily Press; 2012.

[23] Ulbricht T, Southgate D. Coronary heart disease: seven dietary factors. Lancet. 1991; 338(8773):985–992. 10.1016/0140-6736(91)91846-m 1681350

[24] Santos-Silva J, Bessa R, Santos-Silva F. Effect of genotype, feeding system and slaughter weight on the quality of light lambs: II. Fatty acid composition of meat. Livest. Prod. Sci. 2002; 77(2–3):187–194.

[25] Ackman R. Seafood lipids and fatty acids. Food Rev. Int. 1990; 6(4):617–646.

[26] Zhang B, Zhang M, Dai F, Jin X. The biochemical compositions and energy content of 16 important resource species in the central and southern Yellow Sea. Mar. Fish Res. 2008; 29(5):11–18.

[27] Usydus Z, Szlifder-Richert J, Adamczyk M. Variations in proximate composition and fatty acid profiles of Baltic sprat (*Sprattus balticus*). Food Chem. 2012; 130(1):97–103.

[28] Zhang Z, Liu L, Xie C, Li D, Xu J, Zhang M, et al Lipid contents, fatty acid profiles and nutritional quality of nine wild caught freshwater fish species of the Yangtze Basin, China. J. Food Nutr. Res. 2014; 2(7):388–394.

[29] Murillo E, Rao K, Durant AA. The lipid content and fatty acid composition of four eastern central Pacific native fish species. J. Food Compos. Anal. 2014; 33(1):1–5.

[30] Steffens W. Effects of variation in essential fatty acids in fish feeds on nutritive value of freshwater fish for humans. Aquaculture. 1997; 151(1–4):97–119.

[31] Usydus Z, Szlinder-Richert J, Adamczyk M, Szatkowska U. Marine and farmed fish in the Polish market: Comparison of the nutritional value. Food Chem. 2011; 126(1):78–84. 10.1016/j.chemosphere.2011.09.019 22014661

[32] Rodrigues BL, da Cruz Silva ACV, da Costa MP, da Silva FA, Mársico ET, Conte CA. Fatty acid profiles of five farmed Brazilian freshwater fish species from different families. PloS One. 2017; 12(6):e0178898 10.1371/journal.pone.0178898 28614390 PMC5470675

[33] Santos S, Oliveira A, Lopes C. Systematic review of saturated fatty acids on inflammation and circulating levels of adipokines. Nutr. Res. 2013; 33(9):687–695. 10.1016/j.nutres.2013.07.002 24034567

[34] Chuecas L, Riley J. Component fatty acids of the total lipids of some marine phytoplankton. J. Mar. Biol. Assoc. United Kingdom. 1969; 49(1):97–116.

[35] Pacetti D, Alberti F, Boselli E, Frega NG. Characterization of furan fatty acids in Adriatic fish. Food Chem. 2010; 122(1):209–215.

[36] Wu Z, Qiu S, Yan S, Chen M, Wang M. Study of fatty acid of muscle oil of six pelagic fish in Minnan Taiwan Bank fishing ground. J. Fish. China. 2000; 24:61–66.

[37] Tocher DR. Fatty acid requirements in ontogeny of marine and freshwater fish. Aquac. Res. 2010; 41(5):717–732.

[38] Schneedorferová I, Tomčala A, Valterová I. Effect of heat treatment on the n-3/n-6 ratio and content of polyunsaturated fatty acids in fish tissues. Food chem. 2015; 176:205–211. 10.1016/j.foodchem.2014.12.058 25624225

[39] Liu K. The tendency of biodiversity variation of phytoplankton in Pearl River Estuary: Dalian Maritime University; 2008.

[40] Li C, Qiu X. Progress on Fatty Acids Composition of Marine Microalgae. Biotechnology Bulletin. 2008; 4:63–65.

[41] Hernández-Martínez M, Gallardo-Velázquez T, Osorio-Revilla G, Castañeda-Pérez E, Uribe-Hernández K. Characterization of Mexican fishes according to fatty acid profile and fat nutritional indices. Int. J. Food Prop. 2016; 19(6):1401–1412.

[42] Abouel-Yazeed A. Fatty acids profile of some marine water and freshwater fish. J. Arab. Aquac. Soc. 2013; 8(2):283–92.

[43] Belling G, Abbey M, Campbell J, Campbell G. Lipid content and fatty acid composition of 11 species of Queensland (Australia) fish. Lipids. 1997; 32(6):621–625. 10.1007/s11745-997-0079-z 9208391

[44] Özogul Y, Özogul F. Fatty acid profiles of commercially important fish species from the Mediterranean, Aegean and Black Seas. Food Chem. 2007; 100(4):1634–1638.

[45] Dhaneesh KV, Noushad KM, Kumar TTA. Nutritional evaluation of commercially important fish species of Lakshadweep archipelago, India. PLOS One. 2012; 7(9):e45439 10.1371/journal.pone.0045439 23029011 PMC3448644

[46] Kocatepe D, Turan H. Proximate and fatty acid composition of some commercially important fish species from the Sinop region of the Black Sea. Lipids. 2012;47(6):635–641. 10.1007/s11745-012-3658-1 22322400

